# Ethylene-induced transcriptional and hormonal responses at the onset of sugarcane ripening

**DOI:** 10.1038/srep43364

**Published:** 2017-03-07

**Authors:** Camila P. Cunha, Guilherme G. Roberto, Renato Vicentini, Carolina G. Lembke, Glaucia M. Souza, Rafael V. Ribeiro, Eduardo C. Machado, Ana M. M. A. Lagôa, Marcelo Menossi

**Affiliations:** 1 Departamento de Genética, Evolução e Bioagentes, Instituto de Biologia, Universidade Estadual de Campinas, 13083-862, Campinas, Brasil; 2 Centro de Ecofisiologia e Biofísica, Instituto Agronômico de Campinas, 13001-970, Campinas, Brasil; 3 Departamento de Bioquímica, Instituto de Química, Universidade de São Paulo, 05508-000, São Paulo, Brasil; 4 Departamento de Biologia Vegetal, Instituto de Biologia, Universidade Estadual de Campinas, 13083-862, Campinas, Brasil

## Abstract

The effects of ethephon as a sugarcane ripener are attributed to ethylene. However, the role of this phytohormone at the molecular level is unknown. We performed a transcriptome analysis combined with the evaluation of sucrose metabolism and hormone profiling of sugarcane plants sprayed with ethephon or aminoethoxyvinylglycine (AVG), an ethylene inhibitor, at the onset of ripening. The differential response between ethephon and AVG on sucrose level and sucrose synthase activity in internodes indicates ethylene as a potential regulator of sink strength. The correlation between hormone levels and transcriptional changes suggests ethylene as a trigger of multiple hormone signal cascades, with approximately 18% of differentially expressed genes involved in hormone biosynthesis, metabolism, signalling, and response. A defence response elicited in leaves favoured salicylic acid over the ethylene/jasmonic acid pathway, while the upper internode was prone to respond to ethylene with strong stimuli on ethylene biosynthesis and signalling genes. Besides, ethylene acted synergistically with abscisic acid, another ripening factor, and antagonistically with gibberellin and auxin. We identified potential ethylene target genes and characterized the hormonal status during ripening, providing insights into the action of ethylene at the site of sucrose accumulation. A molecular model of ethylene interplay with other hormones is proposed.

Sugarcane (*Saccharum* spp.) is a monocot adapted to tropical and subtropical conditions with an uncommon ability to store sugars in the culm, where sucrose content may reach 62% of the dry matter^1^. This crop is grown mainly for sugar and ethanol production, but the use of byproducts (i.e., cane tops, bagasse, filter muds, and molasses) for fodder, source of renewable energy, and bioplastics increases its importance worldwide^2^. Cultivated in 106 countries, sugarcane occupies an area of approximately 27 million hectares, with Brazil being the largest producer and responsible for approximately 39% of the world production^3^.

In Brazil, sugarcane is planted when temperature and water availability are adequate for sprouting and initial growth, which happens in late summer and early spring^4^. Until fall, high photosynthetic rates support sucrose translocation to actively growing tissues, where sugars are source of energy and carbon for tillering^4^. The intense vegetative growth fades during the winter, when low temperatures and water deficit trigger maturation^5^. During this phenological phase, sucrose is accumulated in the vacuole of parenchyma cells within internodes^6^. Each internode is a growth independent unit, generating a gradient of sucrose in the culm profile: lower internodes have higher sucrose (mature or ripe), while upper ones have lower sucrose content and are still elongating (immature)^6^. With a leaf attached, internode elongates, develops thicker cell wall, and accumulates sucrose, completing the cycle when leaf sheds^6^. When sucrose concentration in the juice reaches up to 13%, the plant is ready for harvest^5^.

The milling schedule and environmental changes can contribute to a deficient natural ripening, making the use of chemical ripeners crucial. Those chemicals mimic the natural ripening even when environmental conditions are sub-optimal, because they inhibit the growth of the apical meristem and divert the energy allocation to sucrose storing^7^. In fact, the improvement of sugarcane technological quality through chemical management has a positive impact on the profits of the cane-based industry^7^. Ethephon, an ethylene releasing compound, was the first growth regulator (early 1960s) used for crop management and post-harvest quality in a wide range of agricultural, horticultural and forestry species^8^. The effects of ethephon are attributed to the phytohormone ethylene^8^. Endogenous ethylene biosynthesis is stimulated and depend on the effective absorption of the chemical, foliar spray coverage, hormone sensitivity, stress status, and environmental conditions^8^. The application of ethephon in sugarcane has accelerated ripening, increased the overall sugar yield, inhibited flowering, and extended harvesting and milling seasons^7^,^9^. Other growth regulator is the aminoethoxyvinylglycine (AVG), a potent antagonist of ethylene that interferes with the hormone biosynthesis^10^, representing a powerful tool for ethylene research^10^,^11^. Although extensively used to postpone senescence (especially undesired abscission) in ornamental and fruit species, AVG has never been applied to sugarcane fields.

A better understanding of the regulation of sucrose accumulation, biomass production, and stress tolerance represents major breakthroughs for the sugarcane industry in an era of challenges, such as climate change, water scarcity, and high energy demand. While in other species these physiological processes are regulated by the hormone ethylene^8^, its role in sugarcane is still to be unravelled. Thus, the aim of this study was to investigate ethylene-modulated gene expression in a high sucrose yield and mid-late season maturing sugarcane cultivar through the application of ethephon and AVG at the onset of ripening. A comprehensive transcriptome analysis combined to sucrose metabolism parameters and hormone profiling allowed the identification of target genes and the site of ethylene action, as well as the characterization of potential hormonal crosstalk, providing insights into the role of ethylene in sugarcane ripening. A representative model of ethylene action at the molecular level is also proposed. This study advanced our scientific knowledge on ethylene-driven processes and pointed novel targets for future genome-wide assisted-selection and biotechnology to foster the production of elite cultivars.

## Results and Discussion

### Effect of ethephon and AVG on sucrose metabolism

**Figure 1:**
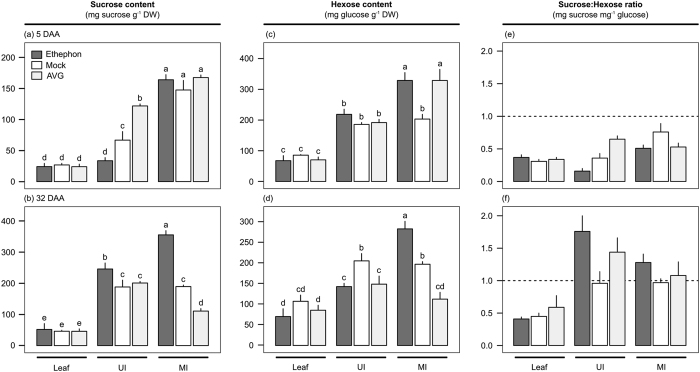
Sucrose (**a**,**b**) and hexose (reducing sugars, (**c**,**d**)) contents (mg sugar g^−1^ dry weight) and sucrose:hexose ratio (**e**,**f**) in leaf and upper (UI, immature) and middle (MI, maturing) internodes of sugarcane plants at five (**a**,**c**,**e**) and 32 (**b**,**d**,**f**) days after ethephon (dark gray), mock (white), or AVG (light gray) application (DAA). Each bar represents the average of four replicates (individual plants), including the standard error of the mean (SEM). The letters denote significant differences using two-way ANOVA (chemical *versus* tissue interaction, p-value < 0.05) followed by LSD test (p-value < 0.05).

**Figure 2:**
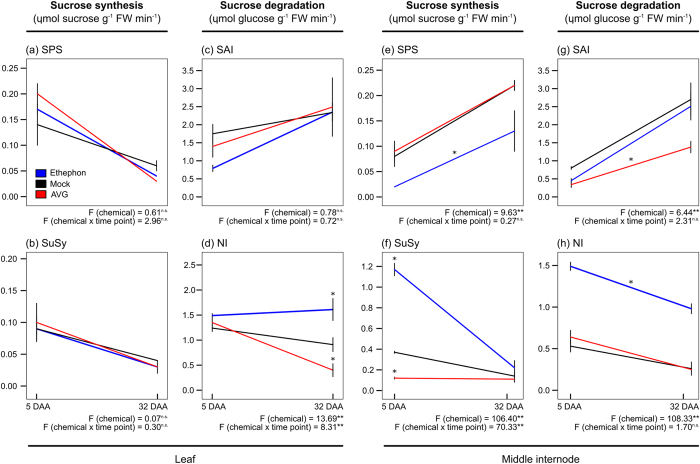
Activity of sucrose metabolizing enzymes in leaf (**a**–**d**) and middle internode (maturing, (**e**–**h**)) of sugarcane plants at five and 32 days after ethephon (blue), mock (black), or AVG (red) application (DAA). The enzymes were involved in sucrose synthesis (*μ*mol sucrose g^−1^ fresh weight min^−1^): sucrose-phosphate synthase (SPS) (**a**,**e**) and sucrose synthase (SuSy, evaluated only at the synthesis direction) (**b**,**f**); and sucrose degradation (*μ*mol glucose g^−1^ fresh weight min^−1^): soluble acid invertase (SAI) (**c**,**g**) and neutral invertase (NI) (**d**,**h**). Each point represents the average of four replicates (individual plants), including the standard error of the mean (SEM). The asterisks denote significant differences between means of growth regulators and mock treatment inferred by one- or two-way ANOVA (F values for chemical and chemical *versus* time point interaction were shown) followed by LSD test (p-value < 0.05 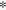 or 0.01*). ^*n*.*s*.^ means not statistically significant.

Ten-month-old sugarcane plants (IACSP95-5000) were sprayed with growth regulators before the onset of ripening: an ethylene-releasing ripener (ethephon), mimicking the standard practices of commercial mills; or an ethylene biosynthesis inhibitor (AVG), hampering putative endogenous ethylene biosynthesis and response. In sugarcane, ethephon stimulates sucrose accumulation in immature internodes accompanied of shoot growth inhibition^7^,^9^. However, to our knowledge, this is the first report about AVG applied at this phenological stage. In sugarcane plantlets grown *in vitro*, AVG antagonized ethephon in promoting tillering and shoot outgrowth^12^, which is a good indication of the opposite effect of both chemicals. Hence, the effect of ethephon and AVG on sugar accumulation was verified through quantification of sucrose and hexose contents on a biomass basis in leaf and culm tissues sampled at the beginning of experimentation (five days after chemical application, DAA) and at harvest (32 DAA) (Fig. 1). Likewise, the activity of key enzymes involved in the regulation of sucrose metabolism was also evaluated to better understand the regulatory role of ethylene on this important process (Fig. 2).

Sucrose and hexose levels on sugarcane leaves were unaltered by growth regulators at both time points (Fig. 1a–d). On the contrary, ethephon-treated canes showed higher sucrose levels in upper and middle internodes (60% more sucrose than mock treatment on average) at harvest, when sucrose:hexose ratio peaked (Fig. 1b,f). Whereas, sucrose content in middle internodes of AVG-treated canes had a 42% reduction when compared to mock treatment (Fig. 1b). Thus, ethephon and AVG were able to modify sugar partitioning in the culm, confirming the efficacy of ethylene in inducing ripening (sucrose storage). The behavior of sucrose metabolizing enzymes was also modulated by chemical spraying (Fig. 2a–h). Enzyme activities were statistically different between chemicals for sucrose-phosphate synthase (SPS, Fig. 2e), sucrose synthase (SuSy, Fig. 2f), and soluble acid invertase (SAI, Fig. 2g) in middle internodes, while neutral invertase (NI) was altered in both leaves and middle internodes (Fig. 2d,h). When considering the interaction between chemicals and time points only NI and SuSy activities showed statistical difference in leaf and middle internode, respectively (Fig. 2d,f).

The magnitude of SPS activity in ethephon-treated internodes (up to 0.03 *μ*mol sucrose g^−1^ FW min^−1^) was low when compared to SuSy (up to 1.33 *μ*mol sucrose g^−1^ FW min^−1^) (Fig. 2e,f). Apparently, SPS activity was reduced by ethephon spraying in relation to mock treatment, while SuSy activity was strongly induced at five DAA in middle internodes. Although, SPS is regarded as a major player in sucrose synthesis in photosynthetic and nonphotosynthetic tissues, with an increased activity towards fully elongated and more mature internodes, its regulation at the protein level by metabolites (e.g., inorganic phosphate) and phosphorylation compromise the enzyme stability and abundance^13^,^14^. SuSy activity shows an opposite behavior of SPS in the culm profile, but its contribution for sucrose synthesis in immature and maturing internodes (from +3 to +9) cannot be underestimated^14^. SuSy has a dual role, catalyzing sucrose synthesis and degradation in a readily reversible reaction, while diverting energy for cell wall formation or ATP-conserving respiration path^15^. The involvement of SuSy in stimulating sink strength has also been speculated for sugarcane as observed in sugar beet root and potato tuber^15^,^16^,^17^. In addition to SuSy, invertases also cleave sucrose, which in parallel with SPS, fine-tune sucrose accumulation through the futile cycle activated whatever internode physiological age (from sucrose mobilising internodes to rapidly storing ones)^18^. Actually, a huge percentage of sucrose (>70%) is hydrolysed prior to storage^6^. SAI activity seemed to be lower than mock treatment in internodes of ethephon-treated canes at five DAA and of AVG-treated canes at both time points (Fig. 2g). On the contrary, NI activity was stimulated in internodes of ethephon-treated canes at both time points (Fig. 2h). There is plenty evidence of SAI activity correlating negatively with internode elongation, however the role of NI is controversial and may be implicated in the control of sucrose flux from vascular to storage tissues in mature internodes^6^,^19^,^20^.

In fact, sucrose storage increases after internodal development, which explains the shoot growth inhibition commonly elicited by chemical ripeners in sugarcane^6^,^7^,^9^. Therefore, low SAI and high SuSy and NI activities just upon ethephon spraying may hasten internode elongation, contributing for the anticipation of sucrose accumulation as described by Fong Chong *et al*.^9^. The modification of enzymatic activity immediately after ethephon application may have a long-lasting effect, culminating in higher sucrose content within internodes at harvest. On the other hand, enzymatic changes detected at harvest may not have a significant contribution to the overall process. We recently found out that IACSP95-5000 accumulated higher sucrose level in culm due to ethephon spraying, even with photosynthesis impaired by drought^21^. This suggests that sink strength modulation, rather than the source activity, is the major ethephon-induced response contributing to sucrose storage. Besides, the differential response of ethephon and AVG to sucrose accumulation and SuSy activity strengthen the ethylene role in sugarcane ripening and should be further investigated.

### Overview of ethylene-induced changes in transcriptome

We performed a transcriptome analysis using tissues from the leaf and the upper internode of plants sprayed with ethephon and AVG at one and five DAA, when the decomposition of ethephon and accumulation of ethylene are pronounced^22^. Equimolar-pooled samples of RNA extracted from three biological replicates (each representing an individual plant) were labelled with either cyanine-3 or cyanine-5 dyes. Co-hybridization of chemical-treated samples with mock samples as reference (one or five DAA chemical treated-sample against one DAA mock sample; and five DAA chemical treated-sample against five DAA mock sample) was performed to monitor gene expression changes in a two-colour Agilent custom microarray, using a dye-swap design (Fig. S1). The twelve chips used in this analysis showed homogeneous distribution of probe intensities, adequate performance of spike-in controls and no significant residual bias associated with dyes (data not shown).

**Figure 3:**
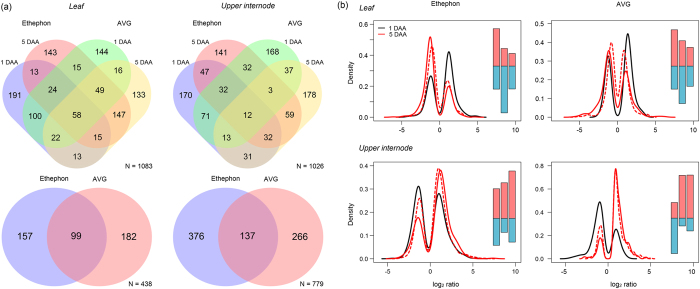
Overview of Sugarcane Assembled Sequences (SAS) identified as differentially expressed (DE) in sugarcane plants sprayed with ethephon and AVG after one and five days (DAA). (**a**) Venn diagrams for leaf and upper internode tissues using one-DAA (top) and five-DAA (bottom) mock samples as reference: N means the number of unique SAS in the diagram. (**b**) Density plots of log_2_ ratio values (the bimodal distribution reflects the selection of DE SAS based on the HTself algorithm), including a bar chart with the number of up (red) and downregulated (blue) genes in the following order (left to right): one (black line) and five (red line) DAA chemical treated-samples against one DAA mock sample (solid line), and five DAA chemical treated-samples against five DAA mock sample (dashed line).

The modified HTself method, with a confidence level of 90% and with the intensity ratio values of replicated probes in the pairwise comparisons consistently assigned outside the credibility interval thresholds (>70%), enabled the classification of genes as differentially expressed (DE)^23^. Among 14,522 Sugarcane Assembled Sequences (SAS) in the array, 2,183 were DE in at least one of the conditions analysed (only sense probes were evaluated) (Table S1), with 394 DE SAS identified on average per array. The majority of DE SAS were uniquely assigned to either ethephon or AVG (approximately 75%), demonstrating that plants exhibited a distinct expression pattern in response to chemicals and time points (Fig. 3a). Additionally, the distribution of log_2_ ratios from each sample, inferred by density plots (Fig. 3b), showed a good agreement between the expression pattern of five-DAA chemical-treated samples using either one DAA or five DAA mock-treated sample as reference (red lines). The number of upregulated SAS in leaves was higher for both chemicals at one DAA (60% more upregulated than downregulated SAS on average), while upper internode samples showed more upregulated SAS at five DAA (190% more upregulated than downregulated SAS on average), with higher expression values (log_2_ ratio > 5, long right tail) for ethephon-treated samples. The gene expression of 17 selected genes obtained by qPCR was suitably correlated (Pearson’s r = 0.6, p-value < 0.002, Spearman’s rank = 0.6, p-value < 0.0001) with those obtained by microarray since the validation was done in a different experimental plot, attesting to the reliability of the data produced (Fig. S2).

### Homology with model species and functional enrichment analyses

**Figure 4:**
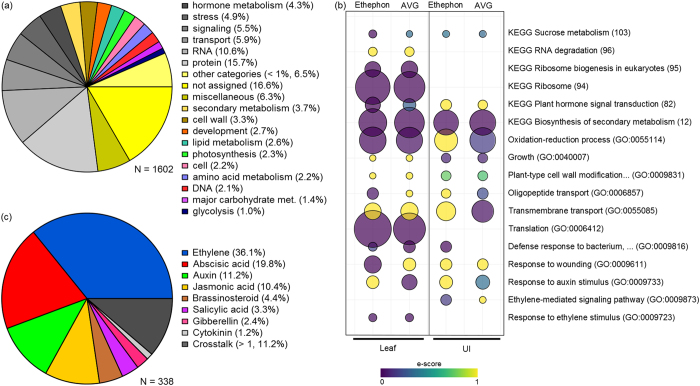
Functional enrichment analyses. (**a**) GOMapMan categories among Sugarcane Assembled Sequences (SAS) identified as differentially expressed (DE) with homology to Arabidopsis. (**b**) Selected statistically significant enriched GO (Gene Ontology) terms (biological process) and KEGG pathways identified in leaf and upper internode (UI) of plants sprayed with ethephon and AVG; the bubble size indicates the frequency of the GO term within the SAS set. (**c**) Proportion of hormone-related DE transcripts in each category.

The putative function of each DE SAS in our study was assigned through orthology with sorghum or rice genes using the data released by Vicentini *et al*.^24^. Among the 2,183 DE SAS, 2,055 were orthologous to sorghum (*Sorghum bicolor*) and 23 were orthologous exclusively to rice (*Oryza sativa*) genes (Table S1). Only 105 SAS were sugarcane-specific, lacking orthology with those grass-related species. We chose to input sorghum and rice coding sequences (complete genome sequence available) in Mercator^25^ to identify homologous genes in the Arabidopsis (*Arabidopsis thaliana*) genome (best hit). A total of 1,906 sequences were assigned to 1,602 unique Arabidopsis Genome Initiative locus identifier (AGI), with 173 monocot-specific sequences (no hits). Hence, we restricted our analysis to conserved genes between monocots and eudicots. We utilized the MapMan ontology (GOMapMan) released in the Mercator output to calculate the percentage of DE SAS within each category. Putative genes encoding proteins involved in or related to hormone metabolism, stress, RNA, signalling, and transport represented a significant portion (approximately 31%) of transcripts (Fig. 4a).

We evaluated overrepresented GO (Gene Ontology) terms within the biological process category that were comparable between ethephon and AVG samples. First, we performed a functional enrichment analysis in AgriGO^26^ that was summarized by REVIGO^27^, using the Arabidopsis genome as reference. The GO terms, response to chemical (GO: 0042221), transport (GO: 0006810), carbohydrate metabolic process (GO: 0005975), and developmental process (GO: 0032502), were enriched in all conditions analysed (p-values ranging from 9.5 × 10^−29^ to 1.6 × 10^−7^). Similar GO terms were found enriched in another analysis performed in GeneMerge^28^ implemented in the SUCEST-FUN database, with sugarcane transcriptome as reference (Fig. 4b). We found response to ethylene stimulus (GO: 0009723) enriched in leaf samples sprayed with ethephon and AVG, although ethylene-mediated signalling pathway (GO: 0009873) was only enriched in ethephon-treated upper internode. Interestingly, response to auxin (GO: 0009733) was exclusively assigned to AVG-treated samples. Other GO terms related to growth (GO: 0009831, GO: 0040007), biotic and abiotic stresses (GO: 0009816, GO: 0009611), and transport (GO: 0055085, GO: 0006857) were also found enriched within our data, including KEGG pathways related to biosynthesis of secondary metabolites, sucrose metabolism and plant hormone signal transduction, which seem to be associated with ethylene response in sugarcane at the maturation stage. Particularly sucrose metabolism and growth related categories are consistent with the physiological effects elicited by ethephon application on sugarcane^7^,^9^.

### Hormone-inducible genes: insights from Arabidopsis homologues

Changes in one hormone often affect other hormone pathways, depending on dosage, developmental and environmental cues that shape their intricate interplay^29^. The functional enrichment analysis points ethylene as a trigger of hormone response pathways in sugarcane, similarly as observed for Arabidopsis^30^,^31^. To unravel the underlying crosstalk between ethylene and other hormones that might occur in sugarcane after spraying of growth regulators, we retrieved hormone-related genes that were DE using the Arabidopsis Hormone Database 2.0^32^, GOMapMan bincodes (e.g. hormone metabolism)^25^, and the data released by Nemhauser *et al*.^30^ and Chang *et al*.^31^ (Table S2). In total, 338 putative genes (17.7% of the whole SAS set with homology to Arabidopsis) were identified as being modulated or participating in at least one hormone pathway with 11.2% of those genes being co-regulated by more than one hormone (Fig. 4c). Genes involved in ethylene, abscisic acid (ABA), auxin (IAA), and jasmonic acid (JA) pathways or response were highly represented (approximately 77.5%) (Fig. 4c).

**Figure 5:**
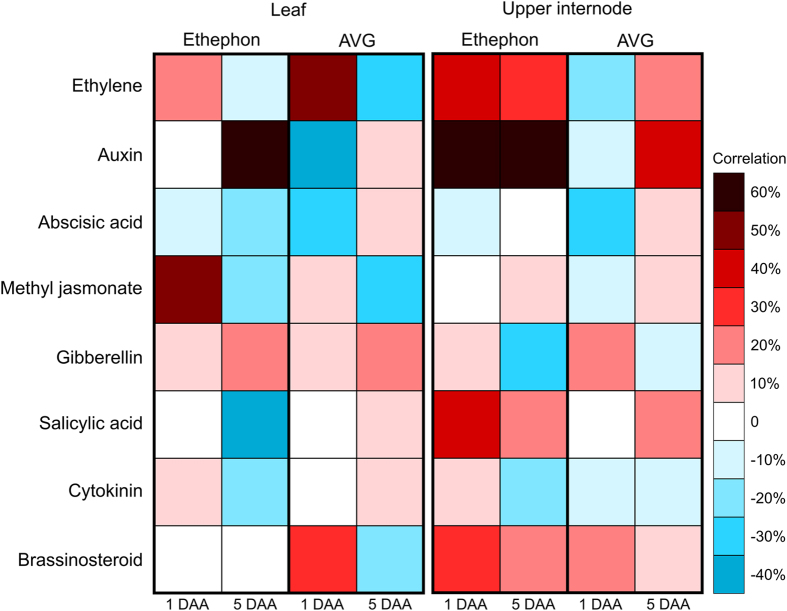
Average correlation values obtained when comparing hormone-related differentially expressed (DE) transcripts identified in sugarcane at one and five days after ethephon and AVG spraying (DAA) with Arabidopsis hormonal transcript indexes (*y* axis) performed in the HORMONOMETER tool^33^. The Arabidopsis hormonal treatments include: ethylene (ACC, ethylene precursor), auxin, abscisic acid, methyl jasmonate, brassinosteroid, cytokinin (zeatin) after 30, 60, and 180 min of exposure; salicylic acid after 180 min of exposure; and gibberellin (isoform 3) after 3, 6, and 9 h of exposure. The range of correlation is colour coded for positive (red), neutral (white) and negative (blue).

The HORMONOMETER tool^33^ was used to correlate multi hormonal signatures compiled from experiments in which seven-day-old Arabidopsis seedlings were subjected to various hormone treatments^34^ with the hormone-related dataset identified in this study (Fig. 5). A strong positive correlation (values above 40%)^33^ with auxin signatures was highlighted in ethephon-treated leaves at five DAA (60% on average) and upper internodes at both time-points (65% on average). On the contrary, IAA signatures showed an anticorrelation with AVG-treated leaves (−40% on average) at one DAA. The 1-aminocyclopropane-1-carboxylic acid (ACC, ethylene precursor) signatures detected in leaf samples had a similar pattern between ethephon and AVG treatments, showing a positive correlation at one DAA and an anticorrelation at five DAA. Otherwise, ACC signatures in the upper internode showed a strong positive correlation with ethephon at both time-points (35% on average) and a weak anticorrelation (−20%) with AVG at one DAA.

### Changes in endogenous hormone levels

**Figure 6:**
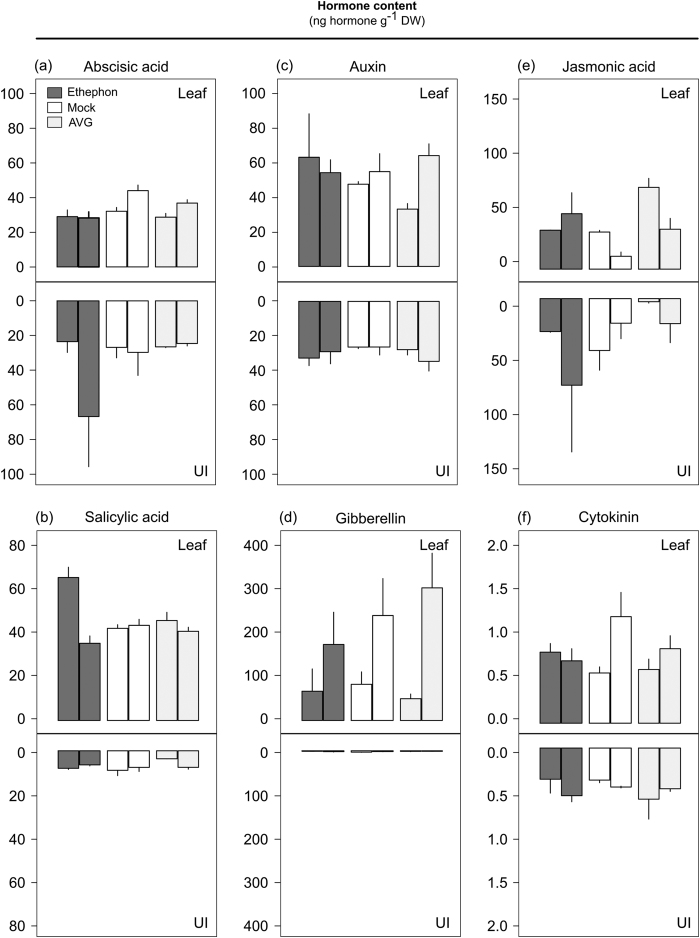
Hormone concentration (ng g^−1^ of dry weight) in leaves and upper internodes (UI) of sugarcane plants treated with ethephon, mock (water), and AVG at one and five days after chemical application (consecutive bars, respectively). (**a**) Abscisic acid (ABA). (**b**) Salicylic acid (SA). (**c**) Auxin (IAA). (**d**) Gibberellin isoform 4 (GA). (**e**) Jasmonic acid (JA). (**f**) Cytokinin, trans-zeatin (CK). Each bar represents the average of four replicates (individual plants), including the standard error of the mean (SEM).

To determine whether growth regulators affect endogenous hormone levels, we quantified ABA, IAA, JA, SA, GA (isoform 4), and cytokinin (CK, *trans*-zeatin) in leaves and upper internodes (Fig. 6) of plants treated with ethephon, AVG, and mock. Although the high variation observed among individual plants impaired the identification of statistically significant differences among chemicals, ethephon seems to affected negatively ABA, GA, and CK levels at five DAA, while SA levels increased substantially at one DAA and JA was slightly higher at five DAA in leaves (Fig. 6a,b,d–f). The ethephon-treated upper internode seems to have higher levels of ABA and JA at five DAA (Fig. 6a,e).

AVG inhibits ACC synthase (ACS), a pyridoxal enzyme that participates in the ethylene biosynthesis pathway, affecting the enzyme activity in a reversible and competitive manner by binding to its catalytic site (K_*i*_ of 0.2 *μ*M)^10^. Besides the known antagonistic response to ethylene, AVG also affects IAA biosynthesis by blocking potentially pyridoxal and tryptophan aminotransferases enzymes throughout its pathway^35^. Therefore, the negative impact of AVG on the endogenous level of IAA in leaves at one DAA, inferred by functional enrichment and HORMONOMETER analyses, is not surprising (Fig. 6c). The AVG dosage used in this study might have elicited a mild AVG response in IAA pathway because its level returned to those observed in the mock treatment at five DAA. Moreover, AVG induced the production of JA in leaves while its level in upper internode seems to decline at one DAA (Fig. 6e).

### Ethylene and other hormone response validation

**Figure 7:**
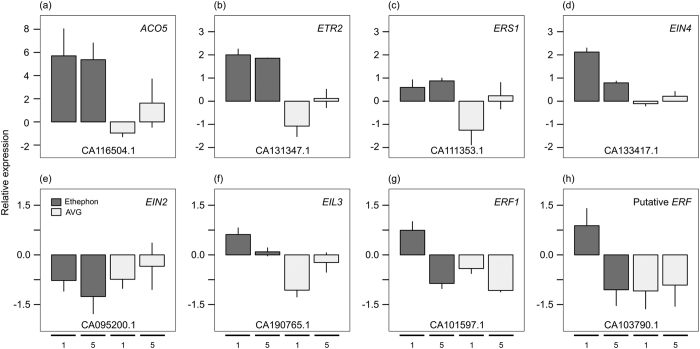
Relative expression of ethylene marker genes in the upper internode of sugarcane plants exposed to ethephon and AVG in relation to mock treatment at one and five days after chemical application (subsequent bars) evaluated by quantitative real-time PCR (qPCR) analysis, using a polyubiquitin gene as reference. Each bar represents the average of three replicates (individual plants), including the standard error of the mean (SEM). The alias was based on orthology to Arabidopsis. NCBI (National Center for Biotechnology Information) accessions are shown at the bottom of each graph.

The effect of ethylene at the molecular level was confirmed using hormone marker genes identified previously^30^,^31^,^32^ (Table S2). The presence of those marker genes confirms ethylene as a modulator of its own biosynthesis, perception, and signal transduction, as well as of other hormone pathways. The ethylene marker genes were mainly responsive to ethephon in upper internode, showing similar expression pattern as reported for Arabidopsis^36^,^37^, rice^38^, *Brachypodium distachyon*^39^, litchi^40^, potato^41^, and rubber tree^42^ upon ethylene gas, ACC, or ethephon exposure. The expression of eight selected ethylene-responsive genes was verified by qPCR in upper internode samples (Fig. 7). Those genes were involved in (i) ethylene biosynthesis: *ACO5* (ACC OXIDASE5, CA116504.1); (ii) perception: *ETR2* (ETHYLENE RECEPTOR2, CA131347.1), *ERS1* (ETHYLENE RESPONSE SENSOR1, CA111353.1), and *EIN4* (ETHYLENE INSENSITIVE4, CA133417.1); and (iii) signal transduction: *EIN2* (ETHYLENE INSENSITIVE2, CA095200.1), *EIL3* (ETHYLENE INSENSITIVE3-LIKE3, CA190765.1), *ERF1* (ETHYLENE RESPONSIVE TRANSCRIPTION FACTOR1, CA101597.1), including a putative *ERF* lacking orthology with Arabidopsis (CA103790.1). The ethylene biosynthetic and receptor genes were induced by ethephon at both time points (Fig. 7a–d) while ethylene signal transduction genes were upregulated at one DAA and downregulated at five DAA (Fig. 7f–h), suggesting a feedback inhibition to fine-tune ethylene response downstream. It is clear that AVG counteracted the effect of ethephon on those genes, showing an opposite expression pattern at one DAA.

**Figure 8:**
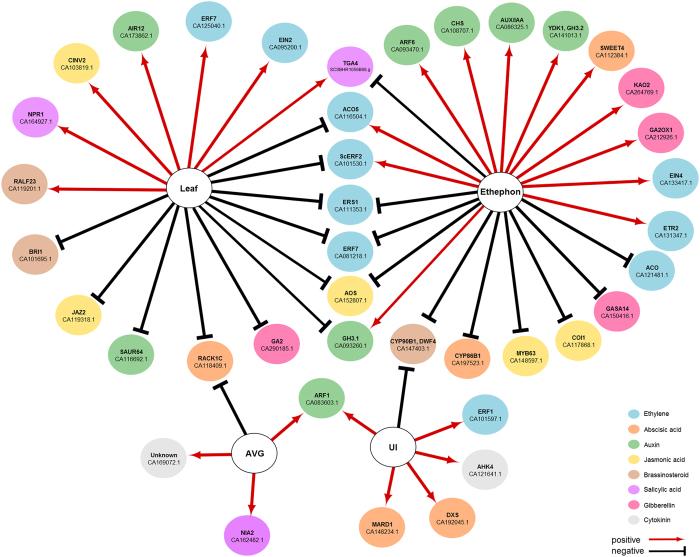
Bayesian network based on the relationship among 60 selected hormone-related genes found to be differentially expressed in the microarray analysis, chemical treatments (ethephon and AVG) and plant tissues (leaf and upper internode). NCBI accession is shown for each transcript, with the exception of *TGA4* identified by the Sugarcane Assembled Sequence (SUCEST). It was assumed that gene expression is dependent on the conditions (chemical and tissue), but not the opposite. The type of interactions indicates positive (red) or negative (black) correlation.

A Bayesian network strategy was used to model the relationship among 60 selected hormone-related genes (Fig. 8, Table S3), composed mostly by marker genes in their respective pathways. Chemical treatments (ethephon and AVG) and plant tissues (leaf and upper internode) were added to the analysis as nodes. The resulting model identified 40 genes linked positively or negatively to the factors analysed, with 12 of them best predicting the ethephon treatment (positive correlation): *ACO5, ETR2, EIN4*, and *SCERF2* (putative *ERF*, CA101530.1), as well as genes involved in IAA (*ARF6*, AUXIN RESPONSE FACTOR6, CA093470.1; *CHS*, CHALCONE SYNTHASE, CA108707.1; a putative *AUX*/*IAA* transcriptional regulator, CA086325.1; *GH3*-*1* and *GH3*-*2*, INDOLE-3-ACETIC ACID-AMIDO SYNTHETASE, CA093260.1 and CA141013.1), ABA (*SWEET4*, bidirectional sugar transporter, CA112384.1), and GA pathways (*KAO2*, ENT-KAURENOIC ACID OXIDASE2, CA264769.1; and *GA2OX1*, GIBBERELLIN 2-BETA-DIOXYGENASE1, CA212926.1). Those genes can be used as biomarkers of ethephon treatment in sugarcane. The number of genes correlated specifically with ethephon was higher than that linked to AVG (Fig. 8). Similarly, leaves showed more correlated genes than upper internodes. Interestingly, genes in the ethylene pathway showed a negative correlation (*ACO5, SCERF2, ERS1*, and *ERF7*, ETHYLENE-RESPONSIVE TRANSCRIPTION FACTOR7, CA081218.1) and two genes in the SA pathway have positive correlation (*NPR1*, NONEXPRESSER OF PR GENES1, CA164927.1; and *TGA4*, SCSBHR1056B08.g) with leaves.

### Site of ethylene action: the internode is ready to respond

The ethylene biosynthetic pathway is accomplished in mainly two rate-limiting steps coordinated by ACS (converts SAM, S-adenosylmethionine, to ACC) and ACO (converts ACC to ethylene)^43^. Genes encoding both enzymes belong to multigene families, whose transcription exhibit temporal-spatial pattern depending on external and internal stimuli^43^. The transcriptional regulation of *ACS* and *ACO* influences ethylene emission^43^, particularly the increase in *ACO* expression accompanied by protein accumulation or high enzyme activity is a strong indicator of auto-regulation of ethylene production within a tissue^43^,^44^. Several transcriptome studies reported the induction of *ACO* upon ethylene exposure^30^,^31^,^34^,^36^,^37^,^40^. In our study, *ACO5* was the uppermost induced gene among ethephon-treated upper internode samples (Table S1). The strong and sustained induction of *ACO5* (40-fold change on average) might indicate an endogenous production of ethylene within this tissue (Table S2). Although another *ACO* gene (CA121481.1) was weakly induced in AVG-treated upper internode, different paralogues might have different roles in auto-regulating ethylene biosynthesis^44^ (Table S2). *ACO5* was also detected in leaf samples, showing similar expression pattern in response to ethephon and AVG, as well as, *ACO* that was downregulated upon AVG treatment (Table S2).

The hormone perception or sensitivity within a tissue is as important as its level in the site of action. The ability to monitor ethylene oscillations depends on the presence of receptors bound to the membrane of the endoplasmic reticulum or the Golgi apparatus, which act potentially as negative regulators of the downstream signalling pathway depending on the genetic background^45^. We identified three putative ethylene receptor genes: *ETR2, ERS1*, and *EIN4* being upregulated upon ethephon stimulus in the upper internode, but only *ETR2* was highly induced in both time-points (Fig. 7b–d). The newly synthesized receptors seem to restore ethylene receptor activity, preparing the plants for future hormone boom and/or switching off the signal transmission downstream, and providing an efficient mechanism for system homeostasis^36^,^40^,^45^. Interestingly, the over-expression of *ETR2* was reported to promote flowering delay and carbohydrate accumulation within rice internodes^46^, which is similar to the physiological changes reported for ethephon application in sugarcane^7^,^9^.

The binding of ethylene to receptors deactivates CTR1, allowing EIN2 to be proteolytic cleaved and free to enter the nucleus to modulate the expression of EIN3/EIL (ETHYLENE INSENSITIVE 3/ETHYLENE INSENSITIVE 3-LIKE)^47^. EIN3/EIL activates the so-called ethylene primary response composed of AP2/EREBP and bHLH transcription factors families members^31^,^47^, which were found significantly enriched in our data (Table S4). Besides the role as central and master regulators of the ethylene signalling pathway, EIN2 and EIN3/EIL integrate other hormone pathways (e.g. IAA, CK and ABA)^47^, triggering the hormonal crosstalk commonly observed upon ethylene stimulus^31^. *EIN2* was downregulated, which was also reported for Arabidopsis^30^, while *EIL3* was upregulated by ethephon in this study (Fig. 7e,f).

The expression pattern of AP2/EREBP family genes varied between ethephon and AVG (Table S2). In upper internode samples, *ERF1* (CA101597.1) and *ERF7* (CA193884.1) showed opposite expression patterns between ethephon and AVG. Whereas, another *ERF7* homologue (CA125040.1) and a putative *ERF* (CA130796.1) were similarly downregulated by both chemicals. Ethephon induced the expression of other three putative *ERFs* (CA103790.1, CA124242.1, and CA101530.1). Among them, CA101530.1 was induced at both time points in ethephon-treated upper internode (3-fold change on average). AVG also affected the expression of *ERFs* in the upper internode: inducing (CA081218.1 and CA117046.1) or repressing them (CA117074.1 and CA127856.1). Differently, the majority of *ERFs* showed a similar expression pattern between ethephon and AVG in leaf samples (CA101597.1, CA081218.1, CA193884.1, and CA064867.1), with the exception of *RAP2*-*2* (CA117074.1) and *ERF073* (CA218955.1).

The synergism between ethylene and JA occurs at the transcriptional level with JAZ (JASMONATE ZIM-DOMAIN PROTEIN) inhibiting EIN3/EIL1^31^,^48^. However, when *JAZ* transcription is repressed, EIN3/EIL1 stability increases upon ethylene stimulus^48^. In the nucleus, the co-receptors, COI1 (CORONATINE-INSENSITIVE PROTEIN1) and the transcriptional repressor JAZ perceive JA conjugates (JA-Ile), targeting JAZ for 26S proteasome-dependent degradation and releasing downstream JA responsive genes^48^. The upregulation of *COI1* (CA117868.1) and the downregulation of *JAZ1* (CA155710.1) in ethephon-treated upper internode indicate a promotion in ethylene and JA responses simultaneously. Transcription factors mutually modulated by ethylene and JA were found within our data (Tables S1 and S2). A confirmed example in sugarcane is *SCERF2* (CA101530.1): induced by ethylene (applied as ethephon) and JA alone (10-fold increase) or in combination (20-fold increase)^49^. Other potential transcripts with the dual regulation are *ERF1* (CA101597.1) and *ICE1* (INDUCER OF CBF EXPRESSION1, CA121236.1). The putative high endogenous JA level in the upper internode (Fig. 6e) might also contribute to a strong ethylene response in the upper internode due to the synergy between them.

The ethephon absorption probably occurs in large quantities in leaves, target of the chemical spray, where the cytosolic pH favours the hydrolysis of ethephon into ethylene (gas) and phosphoric acid^50^. The ability of ethephon eliciting ethylene-related responses is controversial because of its non-controllable or quantifiable hydrolysis^22^. However, the presence of ethylene marker genes, including the strong induction of *ACO5*, receptor genes, and downstream elements of the signalling pathway, upon ethephon spraying exclusively assigned to the upper internode, indicates not only that an ethylene-driven response occurred, but also that this tissue is prone to respond to the hormone. Therefore, the transport of ethylene precursor (ACC), ethylene diffusion from leaves or even a small absorption of ethephon within upper internode (presence of wax and lignified/suberized cell walls might represent obstacles) may account for the ethylene response in sugarcane culms. A hierarchical clustering performed with ethylene-related gene set clearly distinguished ethephon and its antagonist (AVG) only in the upper internode, the site of ethylene action (Fig. S3).

### Ethephon induced defence response in leaves

**Figure 9:**
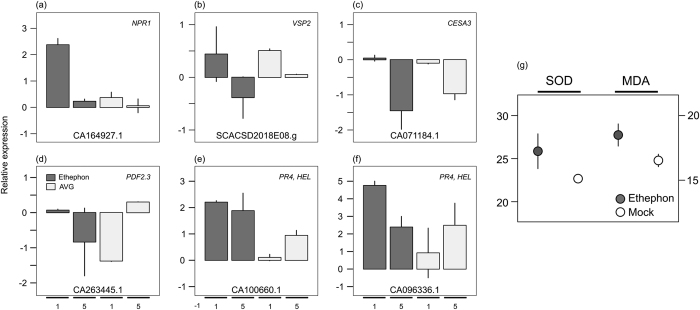
Defence or stress indicators. (**a**–**f**) Relative expression of salicylic acid-mediated suppression of ethylene/jasmonic acid response marker genes in leaves of sugarcane plants exposed to ethephon and AVG in relation to mock treatment at one and five days after chemical application (subsequent bars) evaluated by quantitative real-time PCR (qPCR) analysis, using a polyubiquitin gene as reference. Each bar represents the average of three replicates (individual plants), including the standard error of the mean (SEM). The alias was based on orthology to Arabidopsis. NCBI (National Center for Biotechnology Information) accessions are shown at the bottom of each graph. (**g**) Superoxide dismutase (SOD) activity (units mg^−1^ fresh weight min^−1^) and malondialdehyde (MDA) content (nmol g^−1^ fresh weight) in ethephon and mock-treated leaves evaluated in tissues harvested at five days after chemical spray. Each bar represents the average of six replicates (individual plants), including the SEM.

The defence response in plants is regulated by the interplay of ethylene, JA, and SA^51^. Ethylene and JA act synergistically, while the relationship between JA and SA is often antagonist^51^. However, the fine-tune between the level of hormones and the time of pathway activation is imperative to define which response will be elicited. When the activation of SA signalling pathway occurs concomitantly or before ethylene and JA, the SA response suppresses ethylene/JA signalling components^51^. Firstly, we observed a high level of SA in ethephon-treated leaves at one DAA, not accompanied by an increase in JA level (Fig. 6b,e). Secondly, marker genes of SA-mediated suppression of JA/ethylene pathway were DE, such as: (i) strong upregulation of *NPR1*, a master redox sensor and major activator of SA signalling^52^; (ii) downregulation of genes involved in ethylene/JA-mediated defence, such as a *PDF* (PLANT DEFENSIN, CA263445.1), *VSP2* (VEGETATIVE STORAGE PROTEIN2, SCACSD2018E08.g), and potentially *CESA3* (CELLULOSE SYNTHASE A CATALYTIC SUBUNIT3, CA071184.1); and (iii) reduction in the level of induction of *PR4s* (PATHOGENESIS-RELATED4, CA100660.1 and CA096336.1)^51^,^52^. The expression of those genes was confirmed by qPCR (Fig. 9a–f) and only *CESA3* showed a divergent pattern between microarray and qPCR approaches (Fig. 9c, Table S2).

The production of reactive oxygen species (ROS), mediated by NADPH oxidases and extracellular peroxidases, occurs before the activation of SA pathway^52^. NPR1 requires cell redox alterations to enter the nucleus and activate TGA transcription factors and, consequently, other SA-responsive genes^53^. The activity of superoxide dismutase (SOD), a ROS scavenging enzyme, and the ROS-induced cell damage (inferred by lipid peroxidation through malondialdehyde content) were evaluated in leaf samples treated with ethephon (Fig. 9g). We observed a slight increase in SOD activity in plants exposed to ethephon, when lipid peroxidation seems to be higher than that of mock-treated plants. Coherently, we also identified two NADPH oxidases (*RBOHB*, CA136046.1 and CA132485.1) and two peroxidases (*PER12*, CA104574.1; and *PER52*, CA109102.1) induced in ethephon-treated leaves at one DAA (Table S1). Hence, we speculate that ethephon acting as a stressor or eliciting stressor signals during its decomposition, when acidification may occur, alters the cell redox state with the generation of ROS, which possibly explains the SA burst. Besides, we hypothesized that the prioritization of SA over ethylene/JA response can explain the differential activation of ethylene pathway in leaves and upper internodes after ethephon spraying.

### Growth restrain mediated by auxin and gibberellin deactivation

In this study, no significant differences were observed in IAA content between ethephon and mock treatment (Fig. 6c), although a strong induction of *GH3*-*1* and *GH3*-*2* (19-fold change on average) in leaves and upper internodes treated with ethephon is an indication that the conjugation of IAA was turned on, without visible effects in the time frame evaluated (Table S2). In fact, a decrease in IAA content during sugarcane ripening and senescence has been described^54^,^55^. GH3 and IAGLU (UDP-GLUCOSE:INDOLE-3-ACETATE BETA-D-GLUCOSYLTRANSFERASE) mediate the conjugation of IAA with either amino acids or sugars (UDP-glucose), respectively^56^. Those conjugates control IAA homeostasis through turnover (IAA-Aspartate conjugates) or storing, thereby hampering the hormone action^56^. The low free IAA pool commonly observed during climacteric and nonclimacteric fruit ripening is associated with an increase in IAA conjugates, stimulated by ethylene^57^. Interestingly, GH3 promoted a dwarf phenotype through the control of IAA level in Arabidopsis mutants and rice plants over-expressing *GH3*^58^,^59^. Likewise, the AVG response in leaves seems to be coherent with its inhibitory role on IAA biosynthesis, inferred by a low IAA content (Fig. 6c) and the repression of *GH3*-*1, GH3*-*2* and *AUXIN EFFLUX CARRIERS* (i.e., SCCCAM2004G02.g, CA108976.1, and CA188655.1), including the differential regulation of the majority of *AUX*/*IAA* (auxin-response repressors) identified in this study (i.e., CA093383.1, CA151389.1, CA116567.1, CA102016.1, CA127515.1, CA139092.1) (Table S2).

The low GA_4_ levels detected in culms at the maturation stage (Fig. 6d) was similar as reported by Vasantha *et al*.^54^. The ethephon treatment strongly induced the transcription of *KAO2*, an enzyme that catalyzes the conversion of ent-kaurenoic acid (KA) to gibberellin (GA_12_), precursor of all GA isoforms^60^, as well as *GA2OX1*, which participates in the major route of bioactive GA deactivation (GA_1_ and GA_4_ isoforms), in leaves and upper internodes at one and five DAA. The GA homeostasis is also regulated by DELLA, a potential integrator of ethylene, JA, ABA, GA, IAA, and brassinosteroid pathways^61^,^62^. Ethylene increases the stability of DELLA even in the presence of GA^61^. Among our data, we found an orthologue of *GAI* (GA INSENSITIVE, CA150591.1), one of the DELLA proteins, being slightly induced in the upper internode (Table S2). Additionally, *GASA14* (CA150416.1), responsible for cell elongation and control of ROS accumulation in the presence of GA^63^, was downregulated in the upper internode (Table S2).

IAA and GA are growth-promoting hormones that interfere with sugarcane internode development^64^. High IAA and GA levels concomitantly postpone the senescence process, counteracting the activity of ABA in the apical culm tissue^64^. Therefore, a potential deactivation of IAA (mediated by GH3), and GA (mediated by GA2OX1 and GAI) might lead to shoot growth inhibition and, consequently, affect the timing of sucrose accumulation. Additionally, ethylene stimulates the break of apical dominance (Fig. S4), mainly mediated by IAA inhibition, and flowering delay, through a negative interaction with GA. Those physiological processes are commonly observed in sugarcane upon ethephon spraying^7^,^9^.

### Ethylene and ABA as ripening signals

The induction of *DXS* (1-DEOXY-D-XYLULOSE 5-PHOSPHATE SYNTHASE, CA192045.1) and *NCED9* (9-CIS-EPOXYCAROTENOID DIOXYGENASE9, CA155947.1) in sugarcane culm upon ethephon spraying is a potential indicator of ABA accumulation^65^,^66^ (Table S2). Both enzymes participate in key rate-limiting steps in their respective pathways: DXS is the first enzyme in the methylerythritol phosphate (MEP) pathway, responsible for the production of carotenoids’ 5-carbon precursors (isopentenyl diphosphate, IPP), catalyzing the conjugation of pyruvate and glyceraldehyde-3-phosphate into 1-deoxy-D-xylulose-5-phosphate^67^; and NCED has a pivotal role in ABA biosynthesis, breaking down carotenoids (9′-cis-xanthophylls) to xanthoxin (first ABA biosynthesis intermediate)^66^. Another player in the ABA homeostasis identified as upregulated in this study was *CYP707A1* (ABSCISIC ACID 8′-HYDROXYLASE1, CA162714.1), involved in ABA catabolism^66^. Both genes *NCED9* and *CYP707A1* are known to be transcriptionally activated by ABA^68^.

Cytosolic ABA receptor orthologues (*PYL9*, PYRABACTIN RESISTANCE 1-LIKE 9, CA155779.1 and CA096711.1), known to interact with negative regulators of ABA signalling^69^, were differentially regulated upon ethephon treatment (Table S2). When ABA binds to the receptor complex, the phosphatase activity of protein phosphatase 2C (PP2C) is inhibited, resulting in the activation of ABA response^69^. *CHLH* (MAGNESIUM-CHELATASE SUBUNIT H, CA067637.1, CA103112.1, CA209026.1) is another potential modulator or transducer of ABA signalling^69^, upregulated in our data (Table S2).

Ethylene and ABA interplay modulates the carbon status and, consequently, interferes with photosynthesis and plant growth processes^29^. Besides, those hormones function as ripening factors at the onset of ripening in climacteric and nonclimacteric fruit species, showing non-overlapping peaks, while ethylene biosynthetic genes is induced by ABA and *vice*-*versa*^65^,^70^,^71^. Although ABA accumulation in ethephon-treated upper internodes showed a high variation among biological replicates (16–121 ng g^−1^, Fig. 6a), it is possible that both hormones, ethylene and ABA, function as ripening factors in sugarcane, as well. The ethylene-induced response may stimulate the biosynthesis of ABA, modulating the sucrose accumulation within internodes.

## Concluding Remarks

**Figure 10:**
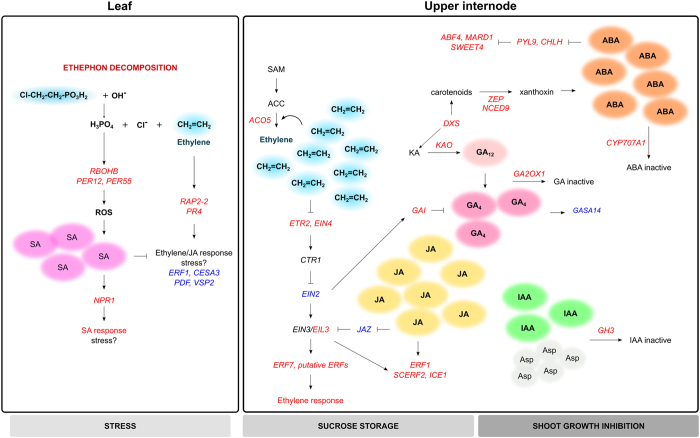
Model of potential crosstalk between ethylene and other hormones upon ethephon spraying in sugarcane plants at the onset of ripening. The ethephon decomposition releases ethylene and phosphoric acid, which may acidify leaf cells. This stress signal might induce NADPH oxidases and peroxidases that stimulate the production of reactive oxygen species (ROS) and, consequently, increase salicylic acid (SA) levels, leading to SA response stimulus over ethylene/jasmonic acid (JA). The ethylene in upper internodes promotes its autocatalytic biosynthesis and transcription of downstream ethylene signalling elements. JA may act synergistically with ethylene, amplifying its response in upper internodes. The synergism between ethylene and abscisic acid (ABA) is also proposed as ethylene seems to promote the expression of ABA biosynthetic genes. Ethylene and ABA may account for sucrose accumulation as ripening signals. The deactivation of gibberellin (GA) and auxin (IAA) through degradation or conjugation might also be induced by ethylene, restraining internode elongation. The genes (italic letters) shown here are placed hierarchically in their respective pathways. Upregulated genes are indicated in red and downregulated genes in blue. The relationship between components include induction (arrow heads) and repression (blocked arrows).

In this study, we provided the first combined ethylene-driven transcriptome and hormone profiling analyses in sugarcane plants at the maturation stage, increasing our general understanding of the ripening process. We showed evidence that a burst in SA level in leaves might have favoured the SA-dependent defence response with the induction of *NPR1*. Markers of SA-mediated suppression of ethylene/JA pathway (e.g., *PDF, VSP2*, and *CESA3*) were downregulated, which may explain the weak ethylene response in leaves and the lack of differences between ethephon and AVG on ethylene-related genes. On the contrary, upper internodes were prone to respond to ethylene: strong induction of biosynthetic and receptor genes indicates a fine-tune of ethylene response, while downstream components (*ERFs*) activate the hormone response. The influence of ethylene on IAA and GA deactivation through induction of *GH3, GA2OX1*, and *GAI* might affect internode elongation, while sucrose accumulation could be modulated by ethylene and ABA, as ripening factors. Moreover, the novel ethylene biomarkers (i.e., *ACO5, ETR2, EIN4, SCERF2, ARF6, CHS* a putative *AUX/IAA, GH3*-*1, GH3*-*2, SWEET4, KAO2*, and *GA2OX1*) identified here can be used to monitor ethylene response in sugarcane in future studies. We devised a hormonal crosstalk model based on our major findings that can provide new directions for novel investigations (Fig. 10).

## Methods

### Plant growth conditions

The experiment was carried out in a greenhouse (open-sided and plastic roofed with a 4 m-height arch structure) at the Santa Elisa Farm, Agronomic Institute of Campinas (IAC), Campinas, Brazil (22°52′6″S, 47°4′32″W) (Fig. S5a). Uniform sugarcane plantlets, sprouted from one-bud cuttings in trays containing commercial substrate, were transplanted in concrete tanks with 1.54 m^3^ of soil (Fig. S5b). The fertilization was based on a soil chemical analysis, following the recommendation of van Raij & Cantarella^72^. Each tank, containing 15 individual plants, represented an experimental unit (Fig. S5c). The whole experiment had six tanks, two per treatment. During plant development, the primary culm was kept, while tillers were periodically removed. The meteorological data inside the greenhouse were recorded with CR800 datalogger (Campbell Scientific, Logan, U.S.A.), where the average air temperature ranged from 16 °C to 29 °C and air relative humidity was about 70%.

### Chemical treatments and plant sampling

The treatments included two growth regulators applied separately: 2-chloroethylphosphonic acid (ethephon), an ethylene releasing compound (Ethrel 240, Bayer CropScience, Charleston, U.S.A.; 519 *μ*M of active ingredient); and aminoethoxyvinylglycine (AVG), an ethylene biosynthesis inhibitor (Retain, Valent BioScience, Libertyville, U.S.A.; 46 *μ*M a.i.). A mock treatment was included, containing only the surfactant (Haiten, Arysta Lifescience, Salto de Pirapora, Brazil; one mL L^−1^ of spray mixture) added to distilled water, as used in the other treatments. The AVG dosage was determined in previous pilot trials using sugarcane cultivars RB966928 and RB867515, in which higher doses than the used here caused leaf chlorosis (data not published). Sugarcane plants that are rapidly storing sucrose at the maturation stage registered a maturation index (ratio between the °Brix at the top and the bottom part of the culm) higher than 0.60, while harvest occurs between 0.85 and 1.00. In this study, chemicals were sprayed when plants were 10-month-old with a maturation index of 0.4, indicating that application occurred prior to ripening. The chemicals were applied with a spray boom with three hollow cone nozzles (TP8002VK, maximum nominal pressure of 20 bar, TeeJet, Springfield, U.S.A.), coupled with an electrical backpack sprayer (model 16 L Jett, Sanmaq, Bragança Paulista, Brazil) at the maximum flow rate (0.8 L min^−1^) and with a total volume of 16 L (Fig. S5d,e). Prior to the application, a plastic curtain divided the area of each treatment to minimize drift. The first leaf with visible dewlap (leaf +1) or the following downward leaf (leaf +2), and the pith (mainly storage parenchyma cells) of the intermediate internode of the upper third (from +2 to +4) and middle third (from +7 to +9) of the culm (here referred as upper internode and middle internode, respectively) of three individual plants per tank were sampled at one, five and 32 days after chemical application (DAA), always at the midday. A total of three individual plants per tank (six per treatment) per time point was harvested; the tissues of interest were separated, immediately frozen in liquid nitrogen, and maintained at −80 °C for further molecular and biochemical analyses. The plants sampled from tanks with the same treatment were harvested and processed in parallel.

### Total RNA extraction and cDNA synthesis

The frozen samples were ground using a pre-cooled mortar and pestle for leaves and an electrical homogenizer for internodes. Total RNA extraction from leaves and internodes was performed using the protocol described by Logemann^73^. The RNA samples were treated with DNase in solution and purified on-column using the RNase-Free DNase Set (Qiagen, Hilden, Germany), according to the manufacturer’s instruction. The integrity of the RNA extracted was assessed by formaldehyde agarose gel (1.2%) electrophoresis and quantified by Nanodrop 2000 (Thermo Scientific, Life Technologies, Waltham, U.S.A.). Absence of contaminant DNA in RNA samples was checked by running a PCR for a promoter sequence. The RNA from three individual plants harvested from one tank were equimolar pooled and used in the microarray as two technical replicates. The remaining plants, from the other tank, were used separately to data validation using quantitative real-time PCR (qPCR). First-strand cDNAs were synthesized from 0.5 *μ*g of purified total RNA in a 20 *μ*L-final volume reaction using QuantiTect Reverse Transcription kit (Qiagen, Hilden, Germany), according to the manufacturer’s instruction. The samples were diluted 1:30 and aliquots were separated to avoid multiple thaws and maintained at −20 °C.

### Microarray procedures

#### Oligoarray hybridization and image acquisition

The customized 4 × 44 K oligoarray (Agilent Technologies, Santa Clara, U.S.A.) for sugarcane (CaneRegNet) contains 21,901 unique probes in duplicates, representing 14,522 SAS released by the Sugarcane EST Project (SUCEST)^74^ and designed as described by Lembke *et al*.^75^. All the steps (from sample preparation to hybridization) followed the Two-Color Microarray-Based Gene Expression Analysis protocol. Spike-in RNA that hybridize to control probes in the array was added to monitor the workflow (Two-Color RNA Spike-In Kit, Agilent Technologies, Santa Clara, U.S.A.). Amplification and labelling (cyanine 5-CTP and cyanine 3-CTP dyes) of 2 *μ*g of total RNA to generate cRNA were performed using Quick Amp Labelling kit, two-color (Agilent Technologies, Santa Clara, U.S.A.). Then, cRNA was purified by RNeasy Mini kit (Qiagen, Hilden, Germany) and quantified in NanoDrop ND-1000 UV-VIS spectrophotometer (Thermo Scientific, Life Technologies, Waltham, U.S.A.). Fluorescently-labelled cRNA was hybridized to arrays using the Gene Expression Hybridization kit and the Gene Expression Wash Buffer kit (Agilent Technologies, Santa Clara, U.S.A.). The arrays were scanned by GenePix 4000B scanner (Molecular Devices, Sunnyvale, U.S.A.) using Agilent (C or B) Scanner Settings. Microarray data files are deposited at the Gene Expression Omnibus (GEO) public database, series record GSE85489.

#### Normalization and data analysis

Scan data were extracted for gene expression measurements using Feature Extraction software version 9.5.3.1 (Agilent Technologies, Santa Clara, U.S.A.) and the protocol GE2-v5_95_Feb07. After a background signal correction, the data were normalized by a linear method followed by the LOWESS method^76^ to correct intensity-dependent dye biases. Outlier genes were identified by a modified HTself method^23^ with 90% confidence for reference dataset, using the following parameters to define significant differentially expressed (DE) genes in each experimental condition: minimum of 70% of all spots showing a similar pattern and positively flagged in the two technical replicates. Finally, the log_2_ ratio was calculated.

### qPCR analysis

Microarray expression validation was performed by qPCR assay, using a polyubiquitin gene as reference designed by Papini-Terzi *et al*.^77^. Target-specific primers were designed from SAS sequences using Beacon Designer Lite software version 8 (Premier Biosoft International, Palo Alto, U.S.A.), showing acceptable efficiencies by LinRegPCR version 2015.3^78^ and specificity confirmed by melting curves. The list of primers used in this study is available at Table S5. qPCR was performed using the 7500 Real-time PCR equipment and 7500 System software (Applied Biosystems, Life Technologies, Waltham, U.S.A.). The reactions were carried out in a 15 *μ*L final-volume, containing SYBR Green PCR Master Mix (Applied Biosystems, Life Technologies, Waltham, U.S.A.), 3.5 *μ*L of cDNA (diluted 1:30) and a final concentration of 300 mM of each primer. Negative controls (water) were added to confirm the absence of any contaminant. The cycling conditions followed the default program using a melting temperature of 60 °C for all primers. Each gene was evaluated in three biological replicates and two technical replicates. The baseline and quantification cycle (C_*q*_) were determined using the Sequence Detection Software version 1.3 (Applied Biosystems, Life Technologies, Waltham, U.S.A.). The relative gene expression was calculated using the comparative C(T) method^79^, followed by a log_2_ normalization.

### Identification of orthologues/homologues in model species

The orthologues from sorghum (*Sorghum bicolor*) or rice (*Oryza sativa*) for each SAS were assigned using the data released by Vicentini *et al*.^24^. The FASTA sequences from sorghum and rice were retrieved and mapped to the *Arabidopsis thaliana* genome (TAIR 10) using Mercator^25^. GOMapMan was used to classify those SAS, showing homology to Arabidopsis, in plant-specific categories. The orthology with Arabidopsis was confirmed using the Ensembl Plants database (release 30, December 2015) only for the SAS discussed in this study. It is worth mentioning that the Arabidopsis alias were used to identify DE transcripts in our study, as no systematic nomenclature is available for sugarcane.

### Functional enrichment and HORMONOMETER analyses

We conducted a GO term enrichment analysis with AGI using agriGO^26^. The list of GO terms was summarized by REVIGO^27^. Additionally, the GeneMerge software^28^, implemented in the SUCEST-FUN database (sucest-fun.org) and manually curated as described by Lembke *et al*.^75^, allowed the identification of overrepresented categories related to GO biological processes, KEGG pathways, and transcription factors. The hormone-related gene set was retrieved using the Arabidopsis Hormone Database 2.0^32^, GOMapMan bincodes (e.g. hormone metabolism), and the data released by Nemhauser *et al*.^30^ and Chang *et al*.^31^. HORMONOMETER software^33^ was used to compare our data with an indexed set of transcripts identified in multi hormonal studies performed with seven-day-old Arabidopsis seedlings^34^.

### Sugar quantification

Freshly harvested tissues (leaf +2 and the pith of upper and middle internodes) were dried to a constant weight at 60 °C in a drying oven. The extract for total soluble sugars and sucrose quantification was prepared as described by Bieleski & Turner^80^ and van Handel^81^, respectively. The quantification method followed Dubois *et al*.^82^, using glucose and sucrose as standards. The content of reducing sugars was calculated subtracting the sucrose from the total soluble sugars. The sugar measurements were corrected by dry weight (biomass) and expressed as mg sugar g^−1^ dry weight (DW).

### Activity of enzymes involved in sucrose metabolism

The activity of sucrose-phosphate synthase (SPS, EC 2.4.1.14), sucrose synthase (SuSy, EC 2.4.1.13), soluble acid invertase (SAI, EC 2.4.1.25), and neutral invertase (NI, EC 2.4.1.26) were evaluated. The extracts were prepared as described by Grof *et al*.^83^, using 500 mg of freshly ground tissues. The purified extract was used in the enzymatic assays according to Zhu *et al*.^84^, with modifications. The SPS and SuSy assays were performed using 1:1 (v/v) of the desalted extract and the reaction buffer. The SPS reaction buffer was prepared using 200 mM Tris-HCl (pH 7.5), 10 mM MgCl_2_, 2 mM EDTA, 25 mM fructose and 50 mM uridine diphosphate glucose (UDPG). The SuSy reaction buffer was prepared as described for SPS, with the exception of fructose that was replaced by 8 mM fructose-6-phosphate and 40 mM glucose-6-phosphate. The mixture was incubated at 37 °C (SPS) or 30 °C (SuSy). The reaction was stopped after 30 min by adding 100 *μ*L of 30% (v/w) KOH and boiling the mixture for 3 min. The sucrose produced by those reactions was measured as described previously. The SAI and NI assays were performed using 1:1:2 (v/v/v) of the desalted extract, 1 M citrate buffer (pH 4.5 for SAI and pH 7.5 for NI), and 0.24 M sucrose. The mixture was incubated at 37 °C for 30 min. The reaction was stopped by adding 0.2 *μ*L of 3 M Tris base and boiling the solution for 3 min. The hexose content was determined by the Somogyi-Nelson method^85^.

### Superoxide dismutase activity

Superoxide dismutase (SOD, EC 1.15.1.1) activity assay was performed as described by Giannopolitis & Ries^86^. The extract was prepared with 100 mg of pulverized leaf tissue, 2% (v/w) PVPP, and 2 mL 0.1 M potassium phosphate buffer (pH 6.8), containing 0.1 mM EDTA and 1 mM phenylmethylsulfonyl fluoride. After centrifugation (15,000 *g*, 15 min, 4 °C), 300 *μ*L of the supernatant was used in the reaction, containing 46 mM sodium phosphate buffer (pH 7.8), 12 mM methionine, 0.1 mM EDTA, 2 *μ*M riboflavin, 0.1 mM nitrotetrazolium blue chloride (NBT), in a final volume of 3.27 *μ*L. Each reaction was performed in replicates: one set was maintained in the dark and the other was exposed to light (30 W) for 5 min. The absorbance at 560 nm was recorded. One SOD unit means the amount of the enzyme required to inhibit the photoreduction of 50% of NBT.

### Lipid peroxidation

Lipid peroxidation was estimated by malondialdehyde (MDA) content according to Cakmak & Horst^87^. The extract was prepared with 200 mg of pulverized leaf tissue and 1.5 mL 0.1% (w/v) trichloroacetic acid. After centrifugation (10,000 *g*, 15 min, 4 °C), 500 *μ*L of the supernatant was used in the reaction, containing 1.5 mL 0.5% (w/v) thiobarbituric acid. The reaction was incubated in a water bath (90 °C) with constant agitation for 20 min. The reaction was stopped on ice followed by centrifugation (10,000 *g*, 10 min, 4 °C). The reaction was maintained for 30 min at room temperature before recording the absorbances at 532 and 600 nm. The MDA content estimation considered a MDA absorbance coefficient of 155 mM^−1^ cm^−1^.

### Hormone profiling

The tissues from leaves and upper and middle internodes of four individual plants were lyophilized and weighed out with approximately 70 mg. The hormone profiling was analyzed at the Proteomics & Mass Spectrometry Facility (Donald Danforth Plant Science Center, St. Louis, USA). The quantification was conducted in a LC-MS/MS system, composed by a Shimadzu LC interfaced with an AB Sciex 4000 QTRAP mass spectrometer equipped with a TurboIonSpray (TIS) electrospray ion source. The 4000 QTRAP mass spectrometer was tuned and calibrated according to the manufacturer’s instruction. Hormones were detected using MRM transitions, previously optimized using a series of standard samples containing different concentrations of hormones and deuterium-labelled standards. For LC separation, a monolithic C18 column (Onyx, 4.6 mm × 100 mm, Phenomenex) with a guard cartridge was used, flowing at 1 mL min^−1^. The peak area was normalized in the same way as the standard samples and, then, quantified according to the standard curve. The data were normalized based on the internal standards (D2JA, D4SA, D6ABA, D5IAA, and D5 *trans*Z) to account for experimental variation, hormone extraction, and ionization efficiency.

### Bayesian network analysis

The Bayesian network analysis was performed using the BNFinder software^88^. The microarray data from all experimental conditions were used to define interactions in a 64 node network, including 60 hormone-related or marker genes, two growth regulators (ethephon and AVG), and two plant tissues (leaf and upper internode). It was assumed that gene expression is dependent on growth regulators and plant tissues, but not the opposite. The orientation of the regulatory interactions was also inferred (positive and negative). The Bayesian-Dirichlet equivalence scoring criterion was used to devise the network structure for the continuous (raw intensity measures) and discrete (growth regulators and tissues) variables. The network topology was visualized using Cytoscape version 3.4.0^89^.

### Statistical analysis

We performed a one-way ANOVA (chemical) or a two-way ANOVA (chemical *versus* tissue or chemical *versus* time point interaction) depending on the data. When the interaction was declared significant (p-value < 0.05), a least significant difference test (LSD, p-value < 0.05) was used to compare means. The analysis was conducted using the package Agricolae in R software. Other analyses were performed with R packages: corrplot (correlation) and d3heatmap (heatmaps and hierarchical clustering).

### Note

 Photographs were taken by C.P.C. and G.G.R. and drawings/graphs were produced by C.P.C. The person depicted in Fig. S4e is G.G.R., who granted the Nature Publishing Group, a division of Macmillan Publishers Ltd, the permission to publish the image under open access license in all formats (i.e. print and digital).

## Additional Information

**Accession codes:** GEO series record GSE85489.

**How to cite this article:** Cunha, C. P. *et al*. Ethylene-induced transcriptional and hormonal responses at the onset of sugarcane ripening. *Sci. Rep.* **7**, 43364; doi: 10.1038/srep43364 (2017).

**Publisher's note:** Springer Nature remains neutral with regard to jurisdictional claims in published maps and institutional affiliations.

## Supplementary Material

Supplementary Information

Supplementary Dataset 1
