# Supplementary material for: Ethylene-induced transcriptional and hormonal responses at the onset of sugarcane ripening
Source: Sci Rep. 2017 Mar 7;7:43364. doi: 10.1038/srep43364 (PMC5339719; doi:10.1038/srep43364)
Supplement: Supplementary Information [file srep43364-s1.pdf]

Ethylene-induced transcriptional and hormonal responses at the onset of sugarcane ripening

Camila P. Cunha, Guilherme G. Roberto, Renato Vicentini, Carolina G. Lembke, Glaucia M. Souza, Rafael V. Ribeiro, Eduardo C. Machado, Ana M. M. A. Lagôa, Marcelo Menossi

Supplementary information - figures

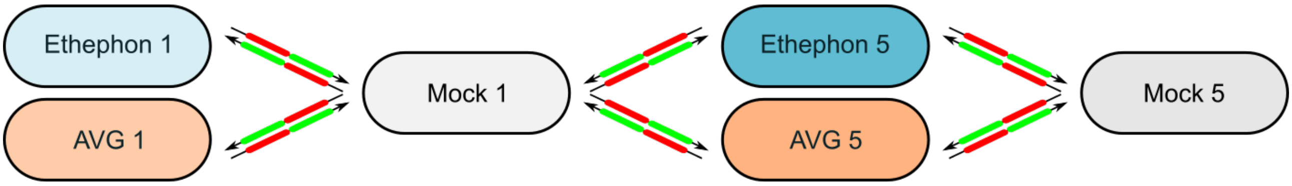

Fig. S1. Experimental design of microarray analysis. The arrows represent different chips in which each sample was labelled with either cyanine-3 or cyanine-5 dyes (here indicated by red and green colours, respectively). The numbers indicate the time point in which samples were harvested: one and five days after chemical application (ethephon, AVG or mock).

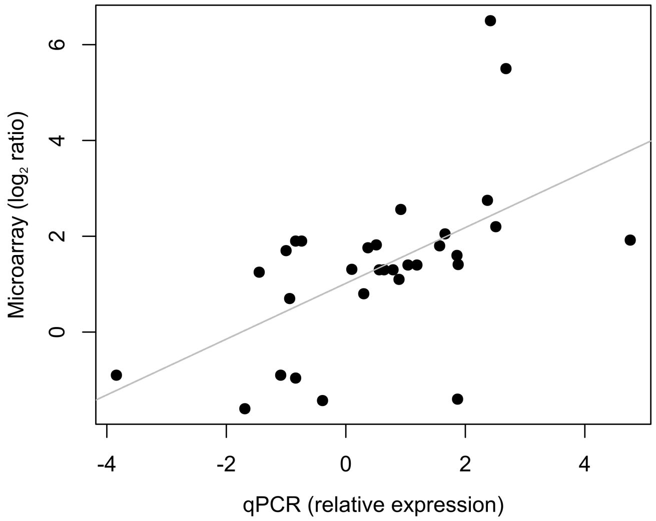

Fig. S2. Correlation between gene expression values of 17 selected genes obtained by microarray and qPCR: Pearson's r = 0.6 (p-value < 0.002) and Spearman's rank = 0.6 (p-value < 0.0001).

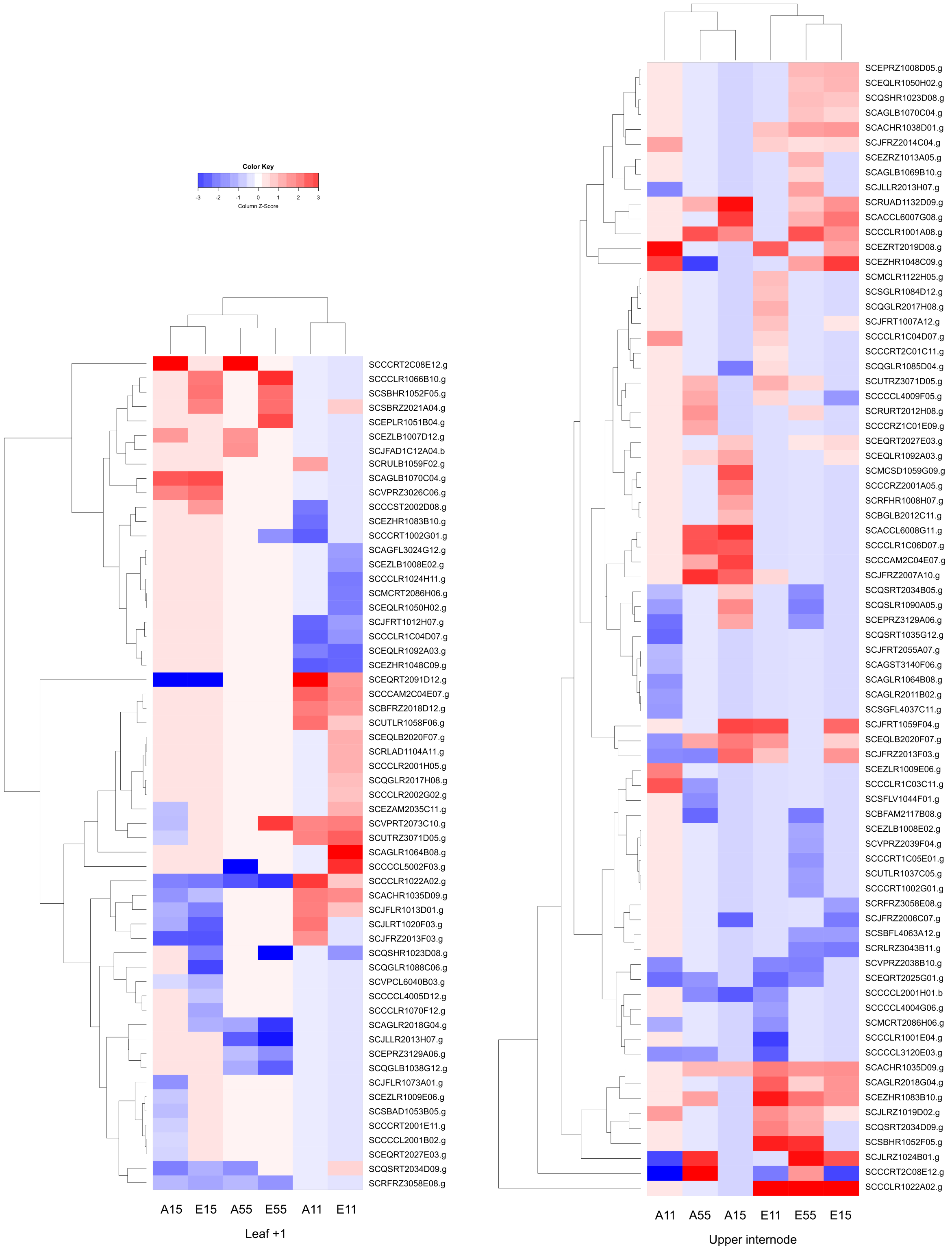

Fig. S3. Hierarchical clustering analysis of Sugarcane Assembled Sequences (SAS) involved in ethylene biosynthesis, perception, signal transduction, and response identified as differentially expressed in leaves (left) and upper internodes (right). Genes and experiments with correlated expression profiles were grouped. The experiments are coded as following: ethephon (E); AVG (A); one DAA chemical treated-sample against one DAA mock sample (11); five DAA chemical treated-sample against one DAA mock sample (51); and five DAA chemical treated-sample against five DAA mock sample (55).

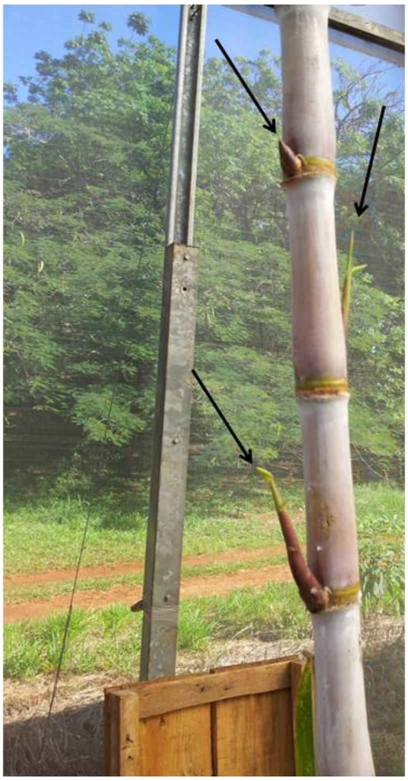

Fig. S4. The break of apical dominance was observed in ethephon-treated plants that remained in the concrete tanks after 32 DAA in this study. The culm shown in the figure was selected as an example.

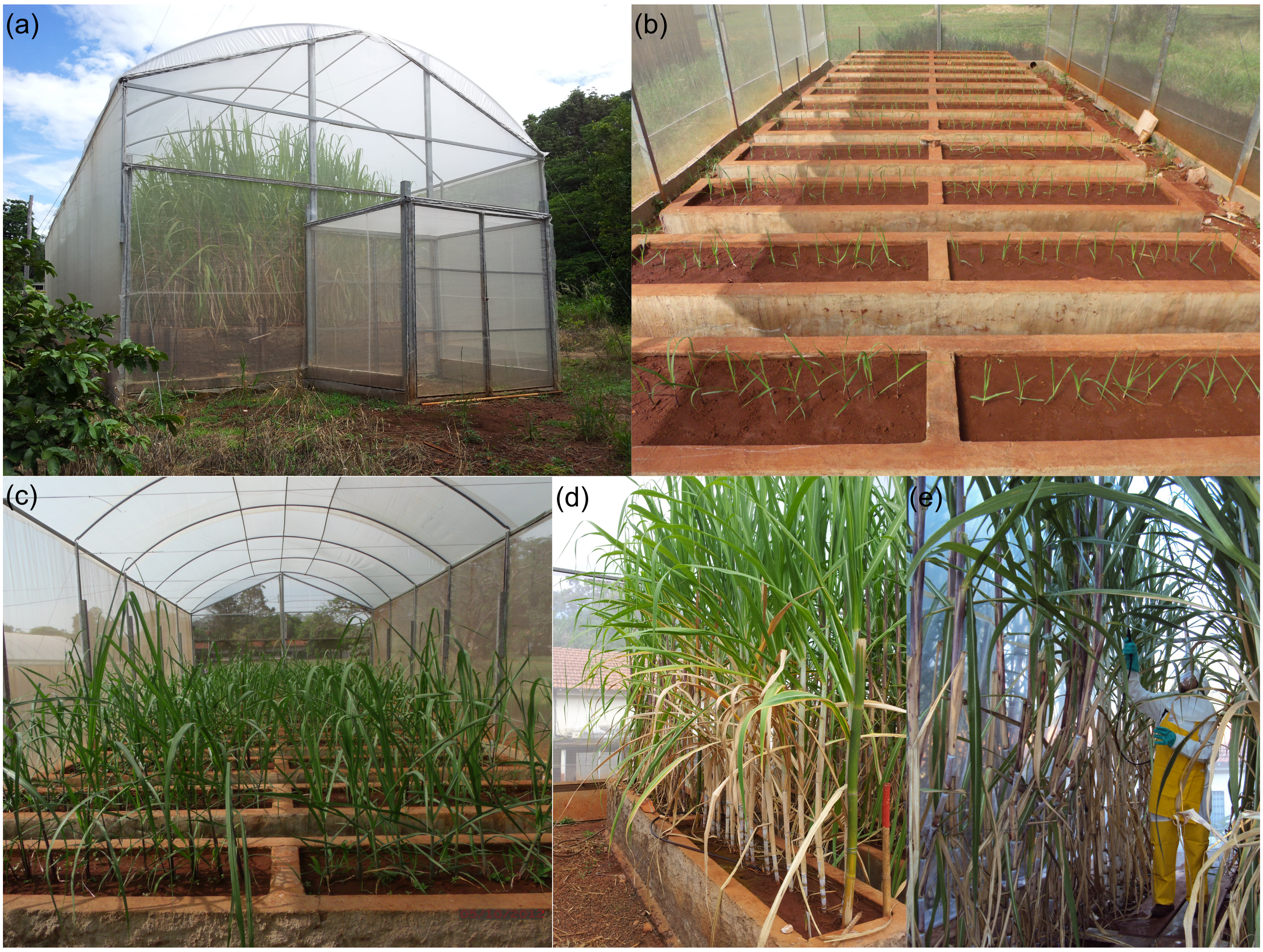

Fig. S5. Greenhouse images. (a) Outside view of the greenhouse. (b) One-month-old plantlets after the transplant to concrete tanks. (c) and (d) Intense vegetative growth stage (four-month-old and eight-month-old plants, respectively). (e) Application of growth regulators using an electrical backpack sprayer occurred when plants reached ten-month-old.

Supplementary Dataset - table legends

Table S1. Whole set of differentially expressed (DE) transcripts identified by microarray analysis (only sense probes are listed). The values represent log_2 ratios. Genes with orthology to model grass species (*Sorghum bicolor*, Sb; and *Oryza sativa*, Os) and homology to *Arabidopsis thaliana* (At) are identified. The experiments are coded as following: leaves (L); upper internodes (UI); E (ethephon); A (AVG). Co-hybridization are coded as following: one DAA chemical treated-sample against one DAA mock sample (11); five DAA chemical treated-sample against one DAA mock sample (51); and five DAA chemical treated-sample against five DAA mock sample (55). ND means not detected as DE.

Table S2. Hormone-related gene set identified among the differentially expressed (DE) transcripts identified by microarray analysis. The values represent log_2 ratios. Genes with orthology to model grass species (*Sorghum bicolor*, Sb; and *Oryza sativa*, Os) and homology to *Arabidopsis thaliana* (At) are identified. ND means not detected as DE; yellow highlight means hormone marker genes used in the Bayesian network analysis; asterisk means hormone marker gene identified in the cited study.

Table S3. Data used for Bayesian network analysis. The raw intensity value for each technical replicate are shown separated by a comma.

Table S4. Transcription factor families significantly enriched in this study. ND means not detected as significantly enriched. Differentially expressed (DE) transcripts from co-hybridizations for the same treatment (ethephon, E; and AVG, A) and plant tissue (leaves, L; and upper internodes, UI) were collapsed.

Table S5. Primers for reference and target genes used in qPCR analysis. The PUB primer, designed by Papini-Terzi et al. 2005 (doi: 10.1093/dnares/12.1.27), was used as reference. The alias was based on orthology to Arabidopsis (detailed in Table S1).
